# The Columbian Exchange as a source of adaptive introgression in human populations

**DOI:** 10.1186/s13062-016-0121-x

**Published:** 2016-04-02

**Authors:** I. King Jordan

**Affiliations:** School of Biology, Georgia Institute of Technology, 950 Atlantic Drive, Atlanta, GA 30332 USA; PanAmerican Bioinformatics Institute, Cali, Valle del Cauca Colombia; BIOS Centro de Bioinformática y Biología Computacional, Manizales, Caldas Colombia

**Keywords:** Columbian exchange, Human evolution, Adaptive evolution, Natural selection, Selective sweep, Genetic admixture, Introgression, Haplotype, Allele

## Abstract

**Background:**

The term “Columbian Exchange” refers to the massive transfer of life between the Afro-Eurasian and American hemispheres that was precipitated by Columbus’ voyage to the New World. The Columbian Exchange is widely appreciated by historians, social scientists and economists as a major turning point that had profound and lasting effects on the trajectory of human history and development.

**Presentation of the hypothesis:**

I propose that the Columbian Exchange should also be appreciated by biologists for its role in the creation of novel human genomes that have been shaped by rapid adaptive evolution. Specifically, I hypothesize that the process of human genome evolution stimulated by the Columbian Exchange was based in part on selective sweeps of introgressed haplotypes from ancestral populations, many of which possessed pre-evolved adaptive utility based on regional-specific fitness and health effects.

**Testing the hypothesis:**

Testing of this hypothesis will require comparative analysis of genome sequences from putative ancestral source populations, with genomes from modern admixed populations, in order to identify ancestry-specific introgressed haplotypes that exist at higher frequencies in admixed populations than can be expected by chance alone. Investigation of such ancestry-enriched genomic regions can be used to provide clues as to the functional roles of the genes therein and the selective forces that have acted to increase their frequency in the population.

**Implications of the hypothesis:**

Critical interrogation of this hypothesis could serve to underscore the important role of introgression as a source of adaptive alleles and as a driver of evolutionary change, and it would highlight the role of admixture in facilitating rapid human evolution.

**Reviewers:**

This article was reviewed by Frank Eisenhaber, Lakshminarayan Iyer and Igor B. Rogozin

## Background

### The Columbian Exchange

**Fig. 1:**
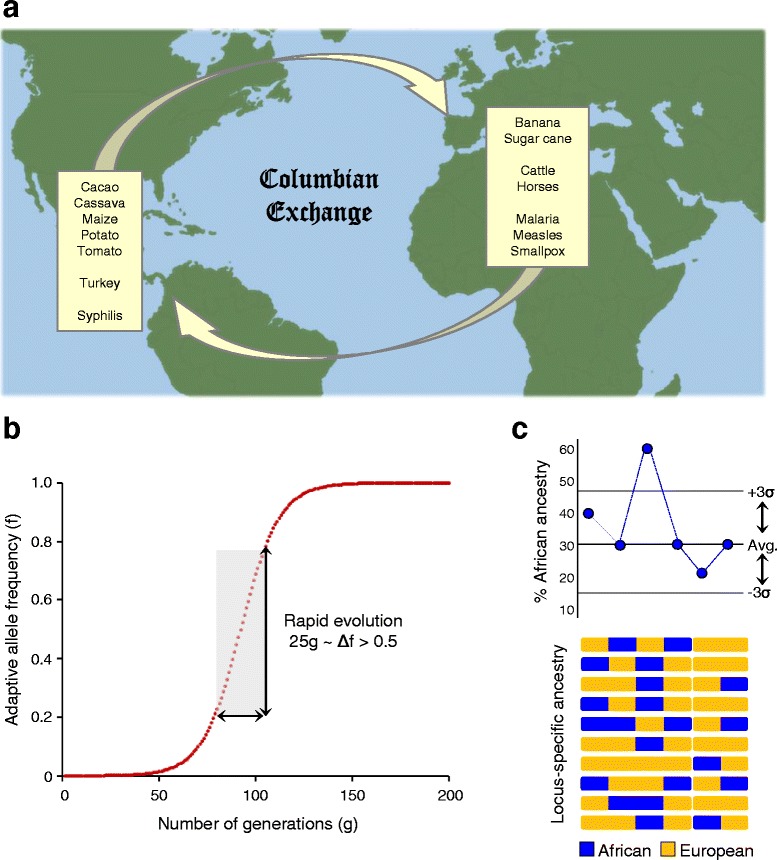
Adaptive introgression via the Columbian Exchange. **a** Examples of plants, animals and microbes transferred between the Old and New Worlds during the Columbian Exchange. Human populations from Europe, Africa and the Americas were also brought together during this era. **b** The number of generations needed to fix an adaptive allele is modeled for a selection coefficient (*s*) of 0.01 and a dominance coefficient (*h*) of 1.0. The level of per-generation adaptive change in allele frequencies varies over four orders of magnitude and reaches its maximum at intermediate allele frequencies. **c** Ancestry-enrichment analysis for the adaptive introgression events. An example is shown for a single chromosome from a hypothetical admixed population with African (avg = 30 %) and European (avg = 70 %) ancestry. Locus-specific ancestry is assigned for all chromosomes in the admixed population, and regions with anomalously high (or low) ancestral origins are identified for further investigation

The historian Alfred Crosby coined the term “Columbian Exchange” to describe the extensive transfer of life between the Afro-Eurasian (Old World) and American (New World) hemispheres following Christopher Columbus’ voyage of 1492 [1]. The Columbian Exchange was a byproduct of subsequent European colonization and trade efforts in the Americas, and it entailed a bidirectional transfer of numerous species of plants, animals and microbes between the Old and New Worlds (Fig. 1a). This transfer also included human population groups, cultures and technologies, and as such it led to major demographic shifts in both hemispheres [2].

The introduction of calorically rich and nutritional New World crop species – potatoes, maize and cassava in particular – facilitated agricultural developments that allowed for sustained population growth in the Old World [3]. The demographic changes in the New World during this era were even more drastic. The Columbian Exchange brought together previously isolated populations from Europe, Africa and the Americas in the New World colonies over a relatively short period of time. More than 50 million Europeans migrated to the Americas through the nineteenth century [4], and the African slave trade resulted in forced migration of 12 million Africans to the New World over a period of ~450 years [5]. The indigenous population of the New World, on the other hand, was reduced by up to 95 % within 100–150 years after Columbus maiden voyage, a loss of an estimated 10–100 million lives [6]; this was largely a result of the introduction of Old World infectious diseases, such as small pox, measles and malaria, to which native populations had little or no resistance. One can expect that such a profound human demographic transformation would occasion substantial evolutionary change at the genomic level.

From a population genomic perspective, the Columbian Exchange can be considered to have facilitated genetic admixture among three human population groups – African, European and Native American – that had previously evolved separately for many thousands of years [7–10]. During the time that these populations were isolated, they accumulated numerous genetic (allele frequency) differences. Many of these differences were likely to be neutral changes with no appreciable effects on fitness, whereas others were the result of adaptations to local selection pressures [11, 12]. In either case, the accumulation of such population-characteristic genetic differences resulted in the presence of distinct haplotypes (*i.e.*, combinations of linked alleles) that are specific to individual populations; the process of admixture during the Columbian Exchange then led to the repeated introgression of these population-specific haplotypes onto distinct genomic backgrounds. In other words, population mixing during the Columbian Exchange generated novel human genome sequences with combinations of haplotypes that had never previously co-existed in the same genome. I am interested in exploring the implications of the rapid creation of such novel, admixed American genome sequences for adaptive human evolution, fitness and health.

### Adaptive introgression and rapid human evolution

As mutation is the ultimate source of novel adaptive alleles, it can be considered to be a critical rate limiting step for adaptive evolution. Human germline mutation rates are low [13], and accordingly adaptive evolution in human populations is generally considered to be a slow process that takes place over many thousands of years [12, 14, 15]. However, introgression can also be an important source of novel alleles for human adaptation [16]. Indeed, a number of recent studies have provided evidence for adaptive evolution of haplotypes that were introgressed from archaic human genomes (Neandertal and/or Denisovan) into modern human genomes [17–20]. Introgression has the potential to speed adaptive evolution by introducing novel alleles at a relatively rapid rate compared to *de novo* mutation.

If genetic admixture between previously isolated populations is extensive, it can provide introgressed haplotypes at intermediate to high frequencies to the resulting admixed population. Introgression could thereby increase the rate of adaptive evolution by elevating the frequency of potentially beneficial alleles available in the population. In this sense, introgression of adaptive alleles can be considered to provide an opportunity for so-called ‘soft’ selective sweeps, which are defined as causing rapid molecular evolution via the simultaneous increase in frequency of multiple adaptive alleles in the population [21]. Soft selective sweeps can occur under several distinct evolutionary scenarios, including the case where multiple adaptive alleles pre-exist in the population as standing genetic variation [22]. Introgression on the scale seen for the three-way population mixing that characterized the Columbian Exchange [7–10] could have provided multiple adaptive alleles as standing genetic variation at intermediate to high population frequencies.

### Presentation of the hypothesis

I hypothesize that genetic admixture and introgression among the human population groups brought together via the Columbian Exchange provided the opportunity for rapid adaptive evolution based on the existence of numerous pre-adapted haplotypes. In other words, introgression during the Columbian Exchange provided extensive standing genetic variation to New World populations, much of it with potential adaptive significance, which could have provided the raw material for numerous (partial) selective sweeps.

The three ancestral human population groups – African, European and Native American – that were brought together over the last 500 years during the course of the Columbian Exchange began to diverge ~60–100,000 years ago (ya) as modern humans emerged from Africa and spread around the world [23]. Europe was populated by anatomically modern humans ~40–45,000 ya [24, 25], and humans reached the Americas ~15,000 ya via several waves of migration across the Bering Strait [26, 27]. As these three population groups were isolated during the course of human evolution, they diverged genetically, accumulating numerous allele frequency differences. A number of these allele frequency differences were likely to be adaptive substitutions with regional-specific utility for health and fitness [11, 12, 15]. These pre-evolved adaptive alleles, and the ancestry-specific haplotypes on which they reside, could have been selected in the admixed American population based on their utility in the New World environment. The process of selection in this case would be based on differential retainment of ancestry-specific haplotypes that provide relatively higher fitness in the admixed population.

The New World environment that served as the selective crucible for the introgressed haplotypes would have consisted of both the external physical environment as well as the internal microbial environment, which was shaped by the mixture of microbes endemic to the ancestral source populations. The novel microbial environment in particular is likely to have exerted strong selective pressure on New World populations, based on the need to respond to the challenge of infectious pathogens, suggesting that immune system genes would be particularly prone to introgression-accelerated adaptive evolution [28–30].

It should be noted that the ~500 years that have elapsed during the era of the Columbian Exchange is an extremely short amount of time with respect to human evolution; indeed, it would correspond to a mere 20–25 generations assuming a generation time of 20–25 years. Irrespective of the role of introgression in providing standing adaptive genetic variation to an admixed population, this would not likely be enough time to allow for the complete fixation of adaptive alleles. Thus, the kind of introgression-facilitated adaptive evolution proposed here would amount to partial (or ongoing) selective sweeps [31]. Nevertheless, substantial levels of allele frequency change can in fact occur over this time scale. A standard model for the rate of fixation of an adaptive allele illustrates how selective increase in allele frequency proceeds successively through slow-fast-slow regimes of change (Fig. 1b). The amount of change per generation is highest at intermediate allele frequencies, where the frequency of a beneficial allele could increase by more than 50 % over 25 generations.

Analysis of human population genetic change over such a relatively short time period provides an opportunity to consider the possibility of rapid human evolution in the context of recent studies related to the convergence of evolutionary and ecological time. The question of extremely rapid evolution, *i.e.* evolution over ecological time scales, has received substantial attention over the last few years [32]. Traditionally, evolutionary and ecological timescales were considered to be very much distinct, but there are numerous studies documenting adaptive evolutionary change that have occurred well within the time span of the Columbian Exchange [33]. However, this line of work has not been explicitly extended to human populations as far as I know, and as such the admixture-introgression conceptual framework outlined here may allow for the evolution-ecology synthesis to incorporate a human dimension.

### Testing the hypothesis

The collection of uniquely admixed human populations found in the Americas represents an ideal laboratory to study rapid human adaptation and to test the hypothesis of adaptive introgression via the Columbian Exchange. To test this hypothesis, one would need to compare genome sequences between putative ancestral populations with sequences characterized from admixed American populations. Comparison with genome sequences from ancestral source populations can be used to characterize the admixture contributions to New World populations at various levels: genome-wide and sex-specific ancestry proportions, local chromosomal ancestry assignments, and ancestry probabilities for individual nucleotide variants (*i.e.*, SNPs) when possible. Having characterized the overall ancestry contribution proportions for an admixed American population, one can then search for specific genomic regions (loci) or SNPs that significantly deviate from the expected patterns (Fig. 1c). This approach can be used to identify genomic loci that are enriched for a particular ancestry, or ancestry-specific haplotypes, suggesting the possibility that such regions were differentially retained in the admixed population based on their utility in the New World environment [7, 28–30, 34–36]. This approach is analogous to the mapping by admixture linkage disequilibrium (or admixture mapping) technique, whereby local deviations from genome-wide average admixture patterns are used to identify loci implicated in diseases that have different prevalences in ancestral source populations [37]. Having identified ancestry-enriched loci (haplotypes) in this way, they can be further interrogated with respect to their rates of evolution as well as the functions of the genes encoded therein.

Interestingly, two studies that were published while this manuscript was in revision both found an excess of African ancestry at the MHC locus in admixed Latin American populations [38, 39]. These results were taken to support the idea of adaptive introgression based on genetic resistance to infectious disease agents, and population genetic modeling was used to provide support for the role of selection in enriching for locus-specific African ancestry. Of course, selection is not the only evolutionary force that could change allele frequencies and lead to the appearance of locus-specific ancestry enrichment. Accordingly, it will be important to explicitly consider the role of other forces, including demography and genetic drift, in shaping patterns of locus-specific ancestry in admixed American populations. Indeed, the need to do so provides an opportunity for the development and application of admixture-specific population genetic models that go beyond the more straightforward sequence-based ancestry enrichment analysis outlined here. The potential for demography to shape patterns of ancestry also underscores the importance of choosing the best possible ancestral population for comparative analysis with admixed American populations. Fortunately, the increasing availability of population genomic sequence data from putative ancestral source populations serves as a rich resource for this purpose.

### Implications of the hypothesis

The hypothesis that the Columbian Exchange facilitated rapid adaptive evolution via admixture and introgression has implications for both basic research in human evolution and for more clinical investigations into genetic determinants of human health. The potential for rapid human evolution is a topic of great interest [40, 41], and critical interrogation of the hypothesis proposed here could help to elucidate one specific mechanism by which such rapid adaptive evolution can be facilitated. It should be emphasized that adaptive evolution predicated upon differential retainment of ancestry-specific haplotypes could entail fairly subtle changes in allele frequencies along the mid-range of the frequency spectrum (Fig. 1b). Thus, it will be expected to occur far more rapidly than complete fixation of new alleles introduced by *de novo* mutation.

Analysis of admixed American populations using this conceptual framework has the potential to reveal human evolution in action. Current methods for detecting the signatures of adaptive evolution (*i.e.*, selective sweeps) in human genome sequences are based on complex statistical models of sequence substitution and may lack power to unambiguously distinguish among different models of selection – *e.g.*, hard versus soft selective sweeps, the role of *de novo* versus standing genetic variation and the prevalence of polygenic selection [22, 31, 42]. Accordingly, there remains substantial controversy as to the relative importance of these different modes of adaptation in human molecular evolution [21, 42]. The alternative introgression-based framework for the detection of potentially adaptive haplotypes that I have outlined here can be considered to be agnostic with respect to these different models of the adaptation process as well as both conceptually straightforward and sensitive to relatively minor changes in allele frequencies [29].

To date, most population genetic studies of New World human populations have focused on ancestry, utilizing sequence variants as neutral markers of evolutionary lineages. Interrogation of the hypothesis proposed here calls for an explicit connection between human genetic ancestry and genetic determinants of health and fitness. The relationship between ancestry and genetic determinants of human health, often manifested as population-specific health disparities [43], is an important topic with serious public health implications. For example, investigation into how admixed populations have been shaped by selection pressures imposed by infectious disease burden can provide insight into the genetic architecture of immune response [44]. Finally, an emphasis on the study of admixed genomes from across the Americas, which testing of the hypothesis articulated here necessitates, could provide for an important extension of current clinical genomic studies, the vast majority of which have focused on populations of European descent [45].

## Reviewers’ comments

### Reviewer 1: Frank Eisenhaber, Bioinformatic Institute, Singapore

The effort of the author to suggest the consequences of the Columbian Exchange for the human genome evolution is laudable and deserves support. The scientific question of how the genomes of many today almost extinct native American peoples survive as admixtures in genomes of European and African origin is of great importance form the evolutionary and historical point of view. Although I do fully agree with the general assessments, conclusions and proposals by the author, I am asking myself whether, at this preliminary point of time, we can speculate plausibly about certain new world characteristics that the natives were better adapted to compared with the arrivals from the other side of the Atlantic Ocean. For examples, have there been any infections/parasites originating from America that represented a major problem for the colonizers and their imported slaves?

Author’s response: *The reviewer raises a very interesting point regarding the threat of infectious disease to European colonizers and African slaves from pathogens native to the Americas. In fact, it is thought that Native American populations had a far lower burden of infectious disease compared to European and African populations. All of humanities major plagues – the black death, cholera, influenza, measles, mumps, smallpox, tuberculosis and typhus – come from the Old World. The relatively low burden of infectious disease among Native American populations has been attributed to the fact that the Americas had far fewer (virtually none in fact) of the domestic animals that serve as sources of zoonotic infections* [46]*, e.g., cows are the natural reservoir for measles, smallpox and tuberculosis, and pigs can harbor whooping cough and flu. The low burden of infectious disease in the Americas, and the related lack of genetic resistance mechanisms, left these populations extremely vulnerable to Old World pathogens and was the major cause of the population crash that occurred during the conquest of the Americas.*

*One possible exception to this pattern is syphilis* [47]*. There is evidence to suggest that syphilis was brought to Europe by Columbus’ returning crew members, and the first recorded epidemic was from Naples, Italy in 1495. Shortly thereafter, syphilis quickly spread throughout Europe with devastating effects. Thus, it may in fact be the case that the burden posed to naïve European and African populations by syphilis in the New World also served as a selection pressure for adaptive introgression of resistant Native American haplotypes.*

The process of admixture as evolutionary process to gain fitness might be contrasted with the consequences of the process of (self-)isolation of populations as it is known, for example, for certain religious groups and heavily stratified societies (see the Indian caste system: “Genomic reconstruction of the history of extant populations of India reveals five distinct ancestral components and a complex structure” Basu et al., PNAS (2016) doi: 10.1073/pnas.1513197113). It would be interesting to study whether at all or to which extent increasingly inbred mating might have detrimental effects on the populations’ fitness.

Author’s response: *This is another interesting point, which is entirely feasible although there is less direct evidence in support of it at this time. It is certainly possible that Native American populations had lower innate (genetic) resistance to infectious disease owing to lower genetic variability associated with bottleneck event(s) that accompanied their initial migration to the Americas from Asia* [48]*. High genetic diversity, among African populations for example, has been associated with increased resistance to infectious disease* [49]*, and low diversity could accordingly place populations at risk. Consistent with the idea raised by the reviewer, our own analysis of Native American populations from the Human Genome Diversity project suggests that they have far lower overall genetic diversity than other populations from this same set of samples and/or from the 1000 Genomes Project. However, this could also be a result of a bottleneck that occurred later as a result of contact with European populations* [50]*.*

### Reviewer 2: Lakshminarayan Iyer, NIH,USA

The article must be published. It is an original idea, untested and likely to provide new insights.

This is a timely, well articulated and well defined hypothesis to test for the presence of partial selective sweeps of haplotypes derived from ancestral populations during the “Columbian Exchange”. It is only in the past 5 years or so that we are even beginning to understand the impact of admixtured populations across various regions and chronological times, leading to fascinating insights on the origins of modern populations, the presence of selective sweeps of alleles and even the impact of these on languages and cultural memes (e.g. PMID: 25731166, 26595274). In this backdrop of development, King’s proposal to test a very specific aspect on the presence of adaptive allele sweeps, from at least 3 or more ancestral populations that contributed to the admixtured populations of the modern Americas, is timely. The idea of using an introgression-based framework is interesting and perhaps quite agnostic as pointed out. Given that both the European and African populations that entered the Americas themselves are derived from multiple ancestral populations, studying the above might further throw light on the evolutionary dynamics of those alleles or loci known to have been selected for in these populations prior to the “Columbian exchange” (e.g. 26595274). I agree that such a study is likely to result in some really interesting insights on the impact of introgression between at least three distinct populations separated by many tens of thousand years.

Author’s response: *I appreciate the positive feedback from the reviewer. I agree that it will be important to take into account the fact that the Europeans and Africans who came to the Americas during the course of the Columbian exchange were themselves derived from multiple (and/or distinct) populations. It should be possible to tease apart the sub-continental admixture contributions from such ancestral populations, and our own group is currently doing so for Afro-Colombian populations in an effort to more precisely locate their origins within the African continent* [51, 52]*.*

*The reviewers’ point also underscores the importance of choosing the most appropriate ancestral populations to perform the kind of ancestry enrichment analysis described here. For example, one may expect that European ancestry for Latino populations would be better modeled using Spanish ancestral populations (e.g., IBS from the 1000 Genomes Project) as opposed to the more widely used CEU sample, which corresponds to broadly distributed Northern European ancestry. Our own preliminary results, suggest that this is in fact the case, and New World admixed populations can be distinguished by virtue of different European ancestry profiles. I have added a paragraph to the “Testing the hypothesis” section of the manuscript to address the need for analysis of appropriate ancestral populations.*

### Reviewer 3: Igor B. Rogozin, NIH,USA

The author hypothesized that the process of human genome evolution stimulated by the Columbian Exchange was based on partial selective sweeps of introgressed haplotypes from ancestral populations, many of which possessed pre-evolved adaptive utility based on regional-specific fitness and health effects. I think that this is a promising hypothesis. The author suggested that in order to test this hypothesis, one would need to compare genome sequences between putative ancestral populations with sequences characterized from admixed American populations.

It is known that natural selection is not the only force that changes allele frequencies, other evolutionary forces include genetic drift, migration, mutation, and biased gene conversion. One potential problem is how to define “putative ancestral populations”, I think that only small (and potentially non-random) samples from putative ancestral populations contributed to the Columbian Exchange (the founder effect). The founder effect might confound the signal of adaptive evolution. However this may not have a substantial impact on the Columbian Exchange, this needs to be evaluated using sequence data although the author may add a brief discussion of this problem to the paper.

Author’s response: *The reviewer raises important points about defining ancestral populations for enrichment analysis and the need to distinguish between the effects of natural selection and demographic processes, both of which can change allele frequencies over time. The issue of the appropriate choice of ancestral populations was also raised by Reviewer #2. Fortunately, there are more and more genomic data sets available from potential ancestral populations, and these data can be compared to genomic data from admixed American populations in order to determine the most appropriate ancestral population for analysis. Nevertheless, the issue of founder effects from ancestral populations, which is entirely reasonable given the history of the conquest and colonization of the Americas, could still confound attempts to quantify changes in allele frequencies by comparing modern populations. This fact, along with the need to distinguish selection from drift present both challenges and opportunities for the development and application of population genetic approaches to this question, in addition to the more straightforward genome sequence analysis approaches described here. I have added a paragraph to the “Testing the hypothesis” section of the manuscript to address the points raised by the reviewer.*

## Abbreviations

SNP: single nucleotide polymorphism
ya: years ago
